# Restoring microRNA-34a overcomes acquired drug resistance and disease progression in human breast cancer cell lines via suppressing the *ABCC1* gene

**DOI:** 10.1007/s10549-023-07170-0

**Published:** 2023-12-07

**Authors:** Shaymaa M. M. Yahya, Heba K. Nabih, Ghada H. Elsayed, Shimaa Ibrahim Abdelmenym Mohamed, Asmaa M. Elfiky, Sohair M. Salem

**Affiliations:** 1 Hormones Department, Medicine and Clinical Studies Research Institute, and Stem Cell Lab, Centre of Excellence for Advanced Sciences, National Research Centre, 33 El Bohouth St., Dokki, P.O. 12622, Giza, Egypt; 2 Medical Biochemistry Department, Medicine and Clinical Studies Research Institute, National Research Centre, 33 El Bohouth St., Dokki, P.O. 12622, Giza, Egypt; 3 Chemistry of Natural and Microbial Products Department, National Research Centre, 33 El Bohouth St., Dokki, P.O. 12622, Giza, Egypt; 4 Environmental and Occupational Medicine Department, Environmental and Climate Change Research Institute, National Research Centre, 33 El Bohouth St., Dokki, P.O. 12622, Giza, Egypt; 5 Molecular Genetics and Enzymology Department, National Research Centre, 33 El Bohouth St., Dokki, P.O. 12622, Giza, Egypt

**Keywords:** MDA-MB-231, MCF-7, Drug resistance, MiRNA-34a, Metastasis, Dual-luciferase reporter assay

## Abstract

**Purpose:**

Breast cancer is one of the leading types of cancer diagnosed in women. Despite the improvements in chemotherapeutic cure strategies, drug resistance is still an obstacle leading to disease aggressiveness. The small non-coding RNA molecules, miRNAs, have been implicated recently to be involved as regulators of gene expression through the silencing of mRNA targets that contributed to several cellular processes related to cancer metastasis. Hence, the present study aimed to investigate the beneficial role and mechanism of miRNA-34a-based gene therapy as a novel approach for conquering drug resistance mediated by ATP-binding cassette (ABC) transporters in breast cancer cells, besides exploring the associated invasive behaviors.

**Material and Methods:**

Bioinformatics tools were used to predict miRNA ABC transporter targets by tracking the ABC transporter pathway. After the establishment of drug-resistant breast cancer MCF-7 and MDA-MB-231 sublines, cells were transfected with the mimic or inhibitor of miRNA-34a-5p. The quantitative expression of genes involved in drug resistance was performed by QRT-PCR, and the exact ABC transporter target specification interaction was confirmed by dual-luciferase reporter assay. Furthermore, flow cytometric analysis was utilized to determine the ability of miRNA-34a-treated cells against doxorubicin uptake and accumulation in cell cycle phases. The spreading capability was examined by colony formation, migration, and wound healing assays. The apoptotic activity was estimated as well.

**Results:**

Our findings firstly discovered the mechanism of miRNA-34a-5p restoration as an anti-drug-resistant molecule that highly significantly attenuates the expression of *ABCC1* via the direct targeting of its 3′- untranslated regions in resistant breast cancer cell lines, with a significant increase of doxorubicin influx by MDA-MB-231/Dox-resistant cells. Additionally, the current data validated a significant reduction of metastatic potentials upon miRNA-34a-5p upregulation in both types of breast cancer-resistant cells.

**Conclusion:**

The ectopic expression of miRNA-34a ameliorates the acquired drug resistance and the migration properties that may eventually lead to improved clinical strategies and outcomes for breast cancer patients. Additionally, miRNA-34a could be monitored as a diagnostic/prognostic biomarker for resistant conditions.

## Introduction

Breast cancer (BC) is the most prevalent cancer type among women and the mainly common malignant tumor in the world among all cancers. BC affects females at any age after puberty in every country around the globe, with rising incidence in later life. In 2020, there were 2.3 million women diagnosed with BC and 685,000 deaths worldwide [1]. According to the glance statistics of the National Cancer Institute, there were 297,790 estimated new BC cases in the year 2023, representing 15.2% of all new cancer cases [2]. Despite the availability of many early detection and treatment options, as well as advances in understanding the molecular mechanisms of BC biology, the disease continues to be fatal and metastatic as a result of rapid development of resistance to cytotoxic medicines and alterations in many gene expressions [3–5]. Consequently as identifying novel effective treatment methods is currently urgent in the field of breast cancer research to improve the clinical management of cancer, adjustment of proliferation, apoptosis, angiogenesis, metastasis, and drug resistance mediatory factors by gene expression regulators has been used as a competence for treatment [6–8].

MicroRNAs (miRNAs) are very important gene expression regulators that have a vital role in controlling many cellular biological processes such as cell growth, proliferation, invasion, differentiation, adhesion, cell death, drug sensitivity/resistance, and cellular metabolic pathways. They are highly conserved naturally occurring small non-coding RNA molecules with a length of about 21–24 nucleotides which can bind partially to their complementary bases in the 3′- untranslated regions (3′UTR) of protein-coding mRNAs, resulting in mRNA translation inhibition or destabilization, leading to gene silencing at the post-transcriptional level [6, 9–11].

MiRNA-34a is one of the miRNA-34 family members that contains three processed miRNAs and are encoded by two different genes. The locus encoding miR-34a is located on chromosome 1p36, while miR-34b and miR-34c share primary transcription from chromosome 11q23. MiR-34a has attracted the greatest attention in the cancer research community because it functions as a tumor suppressor gene; it is also the first miRNA to be shown to be directly regulated by the tumor suppressor p53. MiR-34a is highly expressed in normal cells as compared to carcinomas in which its expression is often downregulated [10, 12, 13]. Because of its critical role in several oncogenic pathways of cancer types, including apoptosis and cell proliferation, miRNA-34a has recently motivated the interest of numerous researchers. Moreover, miRNA-34a has been established to be associated with drug resistance in BC [14–16].

Drug resistance, whether endogenous or acquired, is an important obstacle to cancer treatment success, resulting in an inefficient chemotherapeutic strategy and, ultimately, mortality. There are a number of mechanisms that mediate drug resistance in cancer cells, but the most widely studied one is drug efflux through the overexpressed cell membrane ATP-binding cassette (ABC) transporters that determine the rate of anticancer drugs pumping outside the cells. These drug pumping regulators include P-gp (P-glycoprotein, ABCB1), multidrug resistance-associated protein 1 (ABCC1), ABCC5, ABCC10, ABCF2, and breast cancer resistance protein, ABCG2 (BCRP) [16, 17].

Although several roles of miRNAs in cancer were recognized, the role played by miRNAs in drug resistance is still not fully elucidated [18, 19]. Therefore, the present work aimed to study the therapeutic anti-tumor effects of miRNA-34a restoration in established resistant MCF-7/tamoxifen and MDA-MD-231/doxorubicin cell lines. As well as investigating the mechanism of drug resistance reversal that may be promoted by miRNA-34a upregulation to facilitate the improvement of medicine efficacy and patient response to diverse treatments.

## Material and methods

### Bioinformatics analysis

As described by Kern et al. [20] and Yang et al. [21], the pathway prediction was carried out by miRWALK (http://mirwalk.umm.uni-heidelberg.de) for the ABC transporters investigation using gene-miRNA-pathway analysis tool, the predicted targets were further elucidated by Target scan and RNA22. The mature sequence of miRNA-34a-5p was downloaded from miRBase and the 3'UTR sequences of human ABC transporter genes were downloaded from the Ensembl genome browser.

### Cell lines and cell culture

According to Rana et al. [22], wild MCF-7 (ATCC HTB-22) (human non-invasive luminal type breast cancer) and MDA-MD-231 (ATCC HTB-26) (aggressive human triple-negative breast cancer) human breast cancer cell lines were purchased from ATCC (American Type Culture Collection, VA, USA). Cells were cultured and propagated in 75 cm^2^ flasks in DMEM (Dulbecco’s Modified Eagle Medium; Lonza, Belgium); supplemented with 10% fetal bovine serum (FBS; Biochrom, Berlin), 1% penicillin–streptomycin (Lonza, Belgium), and 4 mM L glutamine (Lonza, Belgium) at 37 ℃ in 5% CO2 incubator. When the cell density reached approximately 80–100%, cells were split for further cultures.

### Establishment of drug-resistant cells

Drug resistance was induced in human breast cancer cells as reported by Krisnamurti et al. [23]. Briefly, MCF-7 and MDA-MB-231 breast cancer cells were treated with tamoxifen and doxorubicin at doses of 1 and 0.18 μM, respectively, for 10 passages. The dramatic upregulation of *ABCB1* and *ABCG* genes to near two or three folds, as well as the morphological changes, confirmed the resistance induction.

### Transfection of cells with microRNA-34a

Hsa-miRNA-34a-5p mimic, inhibitor, and miscript miRNA negative control (NC) were purchased from Qiagen (USA). Both resistant cell lines MCF-7/ tamoxifen (MCF-7/Tx), and MDA-MB-231/Doxorubicin (MDA-MB-231/Dox) were treated with miRNA-34a-5p mimic, or inhibitor, or NC using Hiperfect transfection reagent (Qiagen, USA) according to the following protocol described by Yahya et al. [24]; before transfection, 1.5 × 10^5^ cells were seeded/ well onto two 24-well plates in 500 µl of growth DMEM culture medium. On the second day, cells were transfected with 50 nM miRNA-34a-5p mimic, or inhibitor, or NC in serum-free media using 1 µl of the Hiperfect transfection reagent. After 24 h, the growth media were changed with fresh ones and the cells in the first plate were incubated at the proper experimental conditions for an additional 24 h. The cells in the second plate were subjected to miRNA extraction to verify successful transfection.

### Qiagen miscript system for miRNA-34a determination

MiRNA-34a-5p was determined using the Qiagen miscript system (Qiagen, USA) as directed by the manufacturer. In brief, the miRNeasy kit was used to purify RNA-containing miRNAs, and the miscript RT-kit was used to generate cDNA from the purified RNA-containing miRNAs. In separate procedures, real-time PCR was used to detect mature miRNA utilizing miRNA-specific primers targeting hsa-miRNA-34a-5p. The cycling conditions were as follows: denaturation for 15 s at 94 °C, annealing for 30 s at 55 °C, and extension for 30 s at 70 °C. According to Yahya et al. [24], fluorescence data were obtained using a MiniOpticon Bio-Rad Real-Time Thermal Cycler (France).

### Quantitative real-time gene expression analysis

This was performed using a SYBR^®^ Green one-step RT-PCR kit (Qiagen, USA) after extraction of total RNAs from 1.5 × 10^5^ MCF-7/Tx and MDA-MB-231/Dox cells. The resistant cells treated with miRNA-34a mimic or inhibitor or NC were lysed by QIAzol lysis reagent (Qiagen, USA) according to the manufacturer's instructions. The primer sequences of targeted genes are shown in Table 1. Each gene copy number was normalized to 100,000 copies of the β-actin gene. The RT and subsequent PCR cycling condition was as follow: 50 °C for 10 min, 95 °C for 5 min, 60 °C for 30 s, and then 95 °C for 15 s; the number of cycles was 40 cycles. Bio-Rad Miniopticon™ real-time PCR cycler was used for quantitative estimation [24–26].

**Table 1:** The primer sequences of genes contributed to drug resistance in breast cancinoma

Gene	Forward (5′–3′)	Reverse (5′–3′)
β-actin (housekeeping gene)	CCTTCCTGGGCATGGAGTCCT	GGAGCAATGATCTTGATCTTC
*ABCB1*	AGACATGACCAGGTAT-GCCTAT	AGCCTATCTCCTGTCGCATTA
*ABCC1*	CATTCAGCTCGTCTTGTCCTG	GGATTAGGGTCGTGGATGGTT
*ABCC5*	CTAGAGAGACTGTGGCAAGAAGAGC	AAATGCCATGGTTAGGATGGC
*ABCC10*	GGCTCCGGCAAGTCTTCCCTGTT	AAATGCCATGGTTAGGATGGC
*ABCF2*	TGGAGCAGGGAAGT CAACTCTT	TTTTCGGATCATGCCA TCTG
*ABCG2*	GCGACCTGCCAATTTCAAATG	GACCCTGTTAATCCGTTCGTTT

### Dual-luciferase reporter assay and the vector construction

Referring to Dickson et al. [27], oligonucleotides corresponding to the surrounding predicted target sequence of miRNA-34a-5p in 3' UTR of *ABCB1*, *ABCC1*, and *ABCC5* were designed and cloned into the Dra1 and Xho1 sites of pmirGLO Dual-Luciferase miRNA Target Expression Vector (Promega, USA) which was linearized with the same restriction enzymes then ligated with ligase enzyme. Insertion was verified by PCR (primers and oligonucleotide sequences are listed in Table 2). MCF-7 cells (10,000 cells) were co-transfected with the vector of each construct and miScript negative control (NC treatment), or, mir-34a-5p mimic. After co-transfection for 48 h, the relative luciferase activity was measured using a dual-Glo Luciferase Assay System (Promega, USA). The Dual-Glo® Luciferase Assay System is designed to allow high-throughput analysis of mammalian cells containing genes for firefly and Renilla luciferases grown in 96-well plates. The firefly luciferase luminescence was divided over Renilla luciferase luminescence of each treatment, and then it was calculated as % of control treatment corresponding to its vector construct [21, 28].

**Table 2:** PmirGLO primers and the oligonucleotides used in the dual-luciferase assay

Oligonucleotide	Sequence (5′–3′)
PmirGLOvectorS	ATTAAGGCCAAGAAGGGCGG
PmirGLOvectorS	ATTAAGGCCAAGAAGGGCGG
*ABCB1 S*	*AAA*TGACTTTATCATGAA**ACTGCC**TCATAAATTTGACACCC*C*
*ABCB1A*	*TCGAG*GGGTGTCAAATTTATGAGGCAGTTTCATGATAAAGTCA*TTT*
*ABCC1 S:*	*AAA*CTAGTGACCAAATTCAGCCT**ACTGCC**TCGGATCTCTCCAGCCG*C*
*ABCC1 A:*	*TCGAG*CGGCTGGAGAGATCCGAGGCAGTAGGCTGAATTTGGTCACTAG*TTT*
*ABCC5 S*:	*AAAA*TGACAGGCATAAGCCACTGTA**CACTGCC**TACTTTTTACTTTTTAAATG*C*
*ABCC5 A:*	*TCGAG*CATTTAAAAAGTAAAAAGTAGGCAGTGTACAGTGGCTTATGCCTGTCA*TTTT*

Where ***S*** is the sense strand and ***A*** is the anti-sense strand, MiR-34a-5p recognition sites are in bold, and restriction sites are italic.

### Influx assay

Influx assay was accomplished as stated by Punia et al. [29], with some modifications. Briefly, 2 × 10^6^ MDA-MB-231/Dox cells were transfected with miRNA-34a-5p mimic or NC for 48 h. Then, cells were incubated with 10 µM doxorubicin in phosphate-buffered saline (PBS) for 2 h. Next, cells were harvested, washed, and re-suspended in PBS. Doxorubicin uptake by the cells was examined using a NovoCyte flowcytometry (Agilent, USA) and analyzed by NovoExpress 1.4.1. software. Control background cells were used as a background and its fluorescence measurements were subtracted from all treatment values. 

### Flow cytometric analysis of cell cycle distribution

The DNA content was used to examine cell cycle distribution using the propidium iodide (PI) staining method. Doxorubicin-resistant MDA-MB-231 cells were grown at a density of 1 × 10^6^ cells/ well onto a six-well plate and then transfected with miscript miRNA negative control or miR-34a-5p mimics for 48 h. Cells were then washed with Dulbecco’s phosphate-buffered saline (DPBS), trypsinized using 0.05% trypsin–EDTA, fixed in 70% ethanol in DPBS, and stored at 4 °C overnight. Cells were incubated with 50 µg/ ml PI (Thermo Scientific) containing 8 µg/ml RNase A in the dark at 37 °C for 30 min [24, 26, 30]. Cells were then analyzed by NovoCyte flowcytometer (Agilent, USA). The percentage of cells in G1, S, G2/M and, sub-G1 phases of the cell cycle was calculated using NovoExpress 1.4.1. software.

### Colony formation assay

Both MCF-7/Tx and MDA-MB-231/Dox Cells were cultured for 14 days in 6-well plates with a density of 2 × 10^3^ cells/well after being transfected with miRNA-34a-5p mimics or NC. Colonies were then washed with DPBS, fixed with 1:7 acetic acid/methanol for 5 min at room temperature, washed again with DPBS, dyed with 0.5% crystal violet for 30 min, then washed with tap water and left to air dried [31]. After that, it was captured for counting using an inverted fluorescence microscope (Zeiss, Germany).

### Transwell migration assay

After 48 h of miRNA-34a-5p mimic/ NC transfection, migration assay was performed using polycarbonate membranes with 8-μm pores (Thincert™, Greiner Bio-One, Germany) in 24-well plates. MCF-7/Tx and MDA-MB-231/Dox Cells were serum-starved by incubating cells in media containing 0.2% FBS and then kept at 37 °C in a 5% CO_2_ incubator for 24 h. At a density of 1 × 10^4^ cells/ml in 100 μl of serum-free medium, cells were placed in the upper chamber of the transwell assembly while the lower chamber contained 650 μl of 5% FBS-growth media. After incubation at 37 °C and 5% CO_2_ for 24 h, the upper surface of the membrane was scraped gently to remove non-migrating cells and washed with PBS. The membrane was then fixed in 4% paraformaldehyde for 15 min and stained with crystal violet. The cells were then imaged in five fields for each membrane and counted using Image J software [32].

### Wound healing assay

After miRNA-34a-5p mimic/ NC transfected cells (MCF-7/ Tx and MDA-MB-231/Dox) reached confluency, a 100 µl pipette tip was applied to create a homogeneous scratch wound on the monolayer. Cells were photographed 24 h after creating the scratch. The width of scratches was quantified under an inverted microscope (Zeiss, Germany) at three different positions (bottom, middle, and top), and the mean width was calculated [33].

### Annexin assay for apoptosis and necrosis measurement

These were measured using The RealTime-Glo™ Annexin V Apoptosis and Necrosis Assay (Promega, USA) [34]. Briefly, 10 × 10^3^ cells (MCF-7/ Tx and MDA-MB-231/ Dox)/ well were seeded onto a 96 opaque black plate. The next day cells were transfected with miRNA-34a-5p mimic or NC, and the detection reagent of the RealTime-Glo™ Annexin V Apoptosis and Necrosis Assay which contains Annexin V NanoBiT™ Substrate, CaCl2, Necrosis Detection Reagent, Annexin V-SmBiT and Annexin V-LgBiT reagents was dissolved in cell growth media containing 5% FBS. Six hours after transfection, RLU (Relative Light Unit) and RFU (Relative Fluorescent Unit) were measured using a plate reader (FluoStar, Optima BMG, Germany), after that, FLU (Florescent and luminescent unit) and RFU were recorded at different time intervals for the two consecutive days. A graph was plotted which plots RLU and RFU development against time.

### Caspase 3/7 measurement

The Caspase-Glo^®^ 3/7 Assay (Promega, USA) is a homogeneous, luminescent assay that measures caspase-3 and -7 activities. Briefly, 10,000 cells (MCF-7/ Tx and MDA-MB-231/ Dox) were treated with miRNA-34a-5p mimic or NC for 48 h. After that, a 100 µl of Caspase-Glo^®^ 3/7 Reagent was added to each well, and luminescent was measured using FluoStar, Optima BMG plate reader (Germany) [34].

## Statistical analysis

The results were presented as mean±SEM (standard error of the mean) after their analysis by GraphPad Prism version 5.0 (GraphPad Software Inc., San Diego, CA, USA). A Student’s *t*-test was used to compare individual data points among each group. A *P* value of < 0.05 was set as the criterion for statistical significance. The reproducibility of the data was confirmed as each experiment was performed in triplicate three independent times.

## Results

### Confirmation of miRNA-34a-5p transfection in drug-resistant human breast cancer cells

**Fig. 1:**
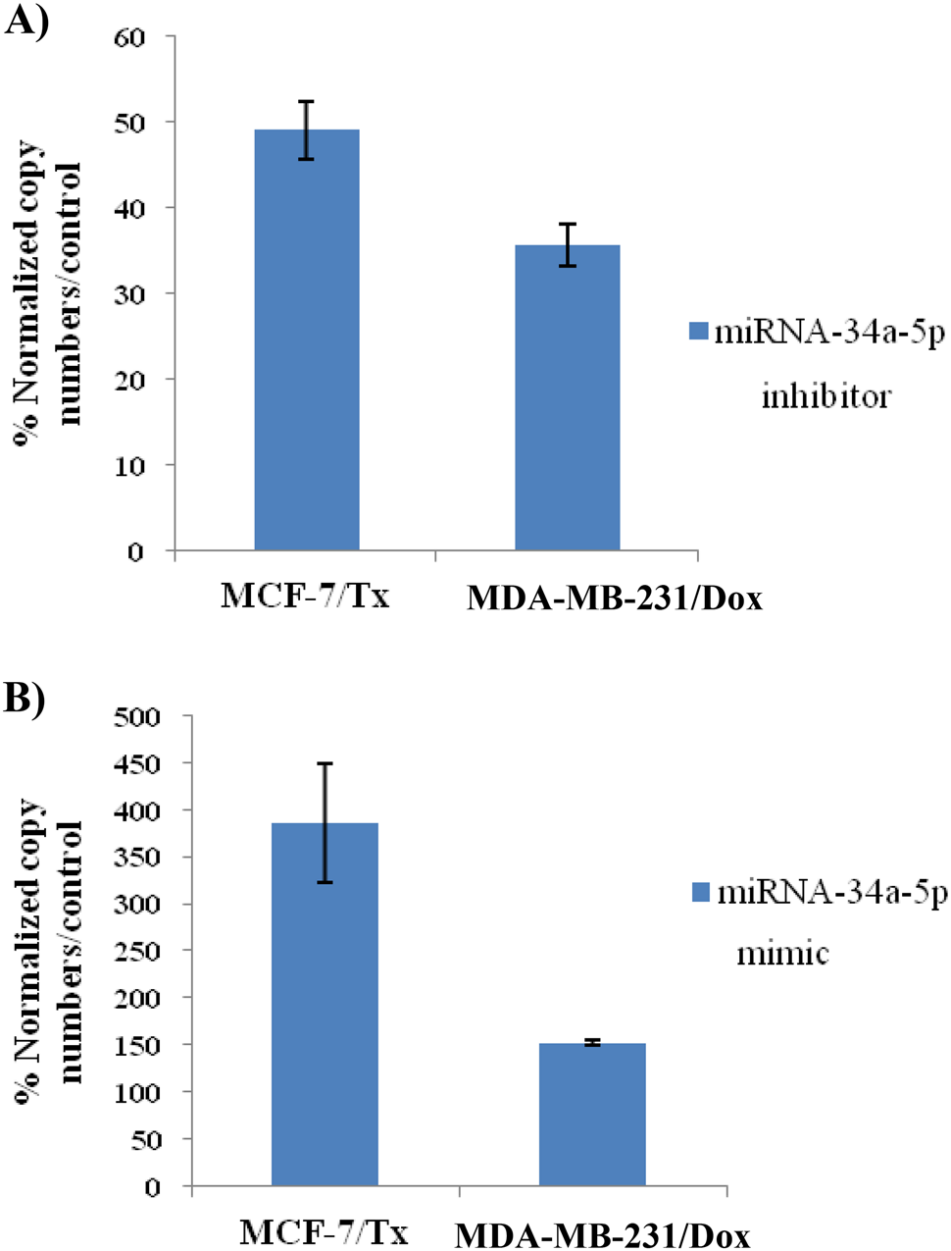
Transfected miR-34a-5p expression levels percentage as compared to NC in cultured human breast cancer-resistant cell lines. **A** The expression level of miR-34a-5p inhibitor in MCF-7-Tx and MDA-MB-231/Dox-treated cells;** B** MCF-7/Tx and MDA-MB-231/Dox cells transfected with miRNA-34a-5p mimic

After transfection of miRNA-34a-5p mimic/inhibitor/NC, miRNA-34a-5p levels were determined in MCF-7/Tx and MDA-MB-231/Dox cells to verify the success of transfection. As evident in Fig. 1A, miRNA-34a-5p expression levels were dramatically decreased in miRNA-34a inhibitor-treated MCF-7/TX and MDA-MB-231/Dox cells as compared to the negative control (NC) treatment. While miRNA-34a-5p expression levels were obviously up-regulated in miRNA-34a mimics-treated MCF-7/TX and MDA-MB-231/Dox cells as compared to the NC treatment (Fig. 1B).

### Verification of the predicted drug resistance-related target genes by Dual-Luciferase reporter assay and QRT-PCR

**Fig. 2:**
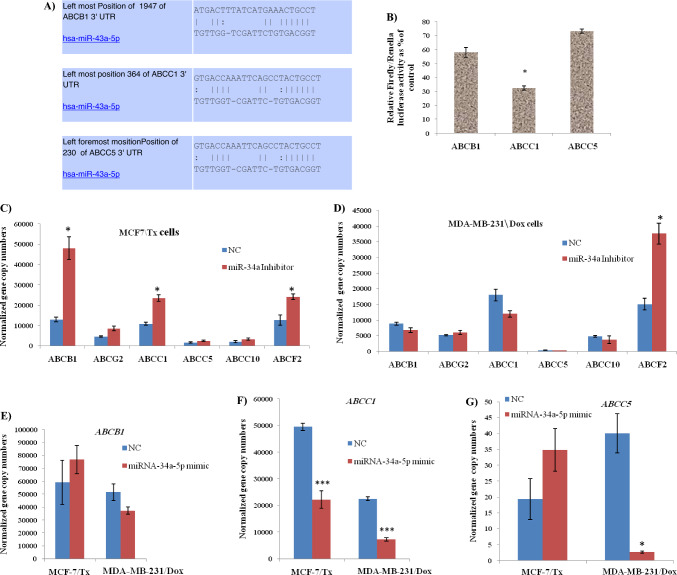
MiRNA-34a-5p targets *ABCB1*, *ABCC1*, and *ABCC5*; **A** Schematic representation of bioinformatics analysis of predicted consequential pairing of ABCB1, ABCC1, and ABCC5 3` UTR targeting sequence and miRNA-34a-5p seed region; **B** Dual-Luciferase reporter assay indicated that MiRNA-34a can bind to 3' UTR of *ABCC1*; **C** ABC transporters gene expression levels in miRNA-34a-5p inhibitor MCF-7/Tx-treated cells; **D** ABC transporters gene expression levels in miRNA-34a-5p inhibitor MDA-MB-231/Dox-treated cells; **E** *ABCB1* transporter gene expression levels in miRNA-34a-5p mimic and NC-treated MCF-7/Tx and MDA-MB-231/Dox cells; **F** *ABCC1* transporter gene expression levels in miRNA-34a-5p mimic and NC transfected MCF-7/Tx and MDA-MB-231/Dox cells; **G** *ABCC5* transporter gene expression levels in miRNA-34a-5p mimic and NC transfected MCF-7/Tx and MDA-MB-231/Dox cells. *P* < 0.05 (*), *** < 0.001

MiRWALK (http://mirwalk.umm.uni-heidelberg.de) analysis using gene-miRNA-pathway analysis tool, Target scan, and RNA22 bioinformatics tools have confirmed that *ABCB1*, *ABCC1*, and *ABCC5* are predicted targets for miRNA-34a (Fig. 2A). To verify this prediction experimentally, in addition to real-time PCR, dual–luciferase reporter assay was conducted. Insertion of the *ABCC1* binding site into the vector and co-transfection with miRNA-34a-5p mimics significantly reduced the luciferase activity, whereas insertion of *ABCB1* and *ABCC5* binding sites into vector and co-transfection with miRNA-34a-5p mimics produced a non-significant decrease in the luciferase activity (Fig. 2B).

As shown in Fig. 2C, miRNA-34a-5p inhibition resulted in a significant upregulation of *ABCB1*, *ABCC1*, and *ABCF2* gene expression levels in MCF-7/Tx cells; while in MDA-MB-231/ Dox cells (Fig. 2D), the expression level of *ABCF2* gene was recorded to be significantly increased as compared to the other genes expression levels.

On the other hand, treatment of MCF-7/Tx and MDA-MB-231/Dox cells with miR-34a-5p mimics revealed a highly significant (*P* < 0.001) decrease in levels of *ABCC1* gene as compared to NC-treated cells (Fig. 2F). Meanwhile, it produced a significant downregulation of *ABCC5* gene expression level in MDA-MB-231/Dox cells (Fig. 2G). However, the changes were insignificant in the *ABCB1* gene as compared to the NC treatment (Fig. 2E).

### Upregulation of miRNA-34a-5p increases doxorubicin uptake by resistant breast cancer cells

**Fig. 3:**
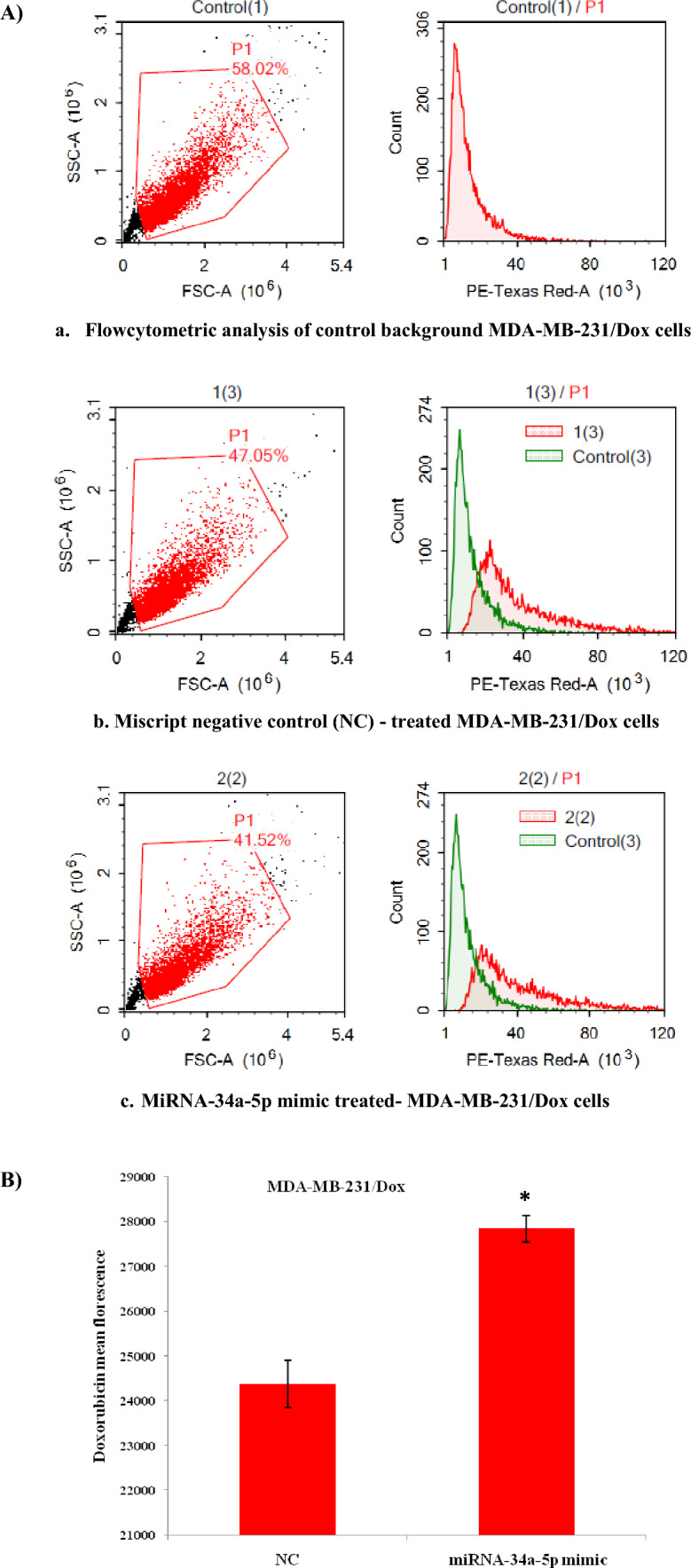
Doxorubicin influx measured by flow cytometry in MDA-MB-231/Dox cells. **A a.** Flow cytometric analysis of control background cells**, b.** Miscript negative control (NC)-treated cells**, c.** MiRNA-34a-5p mimic-treated cells**. B** Doxorubicin mean florescent in cells incubated with miRNA-34a-5p mimic for 48 h. (*P < 0.05)

Doxorubicin influx was detected to be significantly retained in MDA-MB-231/Dox cells treated with miRNA-34a-5p mimics by flow cytometry, in a comparison with NC, as displayed in Fig. 3A and B.

### The effect of miRNA-34a-5p overexpression on cell cycle progression

**Fig. 4:**
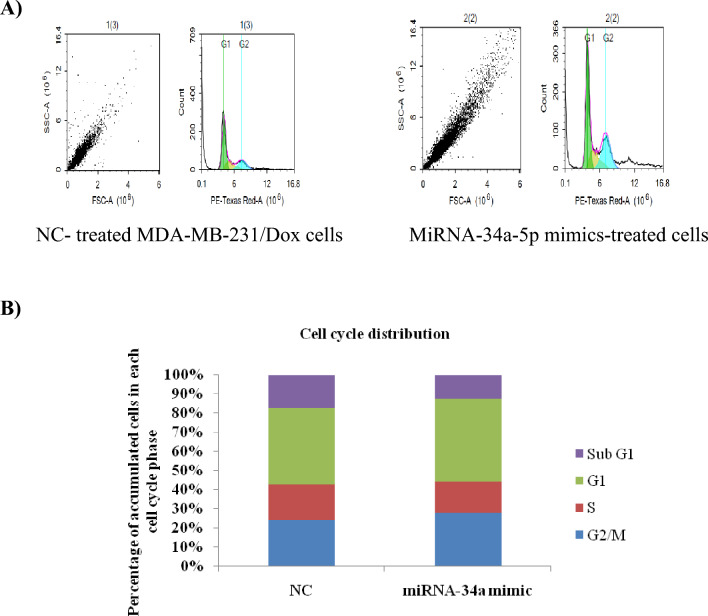
Cell cycle distribution analysis by flow cytometry. **A** Representative data of flow cytometric analysis of MDA-MB231/Dox cells treated with miRNA-34a-5p mimic/NC for 48 h. The analysis was carried out by NovoCyte flow cytometer. **B** A column chart of percentage of accumulated cells in each cell cycle phase as a response to miRNA-34a-5p upregulation in MDA-MB231/Dox cells

As mir-34a-5p downregulation has been observed in many different tumors including breast cancer [35, 36], we increased its concentration within doxorubicin-resistant breast MDA-MB-231 cancer cells by transfection of mir-34a-5p mimics. The results of flow cytometry reported the insignificant effect of this microRNA on cell cycle distribution. The observations noticed a slight increase in the mean percentage of cells in G0/G1 (37 ± 1.07%), and G2/M (23.84 ± 1.8%) phases as compared to the corresponding controls (36 ± 0.82% and 22 ± 2.7%, respectively). At the same time, our results showed a decrease in the accumulation of cells in the sub-G1 phase after 48 h transfection of mir-34a-5p mimics, in a comparison to control (Fig. 4A, B and Table 3).

**Table 3:** Cells accumulation percentage in each phase of cell cycle of MDA-MB-231/Dox cells

Treatment	G1	S	G2/M	Sub G1
NC	36% ± 0.82	16.27% ± 0.96	22% ± 2.7	15.5% ± 6.07
miRNA-34a-5p mimic	37% ± 1.07	14.16% ± 0.23	23.84% ± 1.8	10.47% ± 2.2

The mean percentage of cells in each phase of cell cycle as compared to negative control (NC) treatment

### Malignant biological behavior of miRNA-34a-5p related to progression and invasion in human-resistant breast cancer cells

**Fig. 5:**
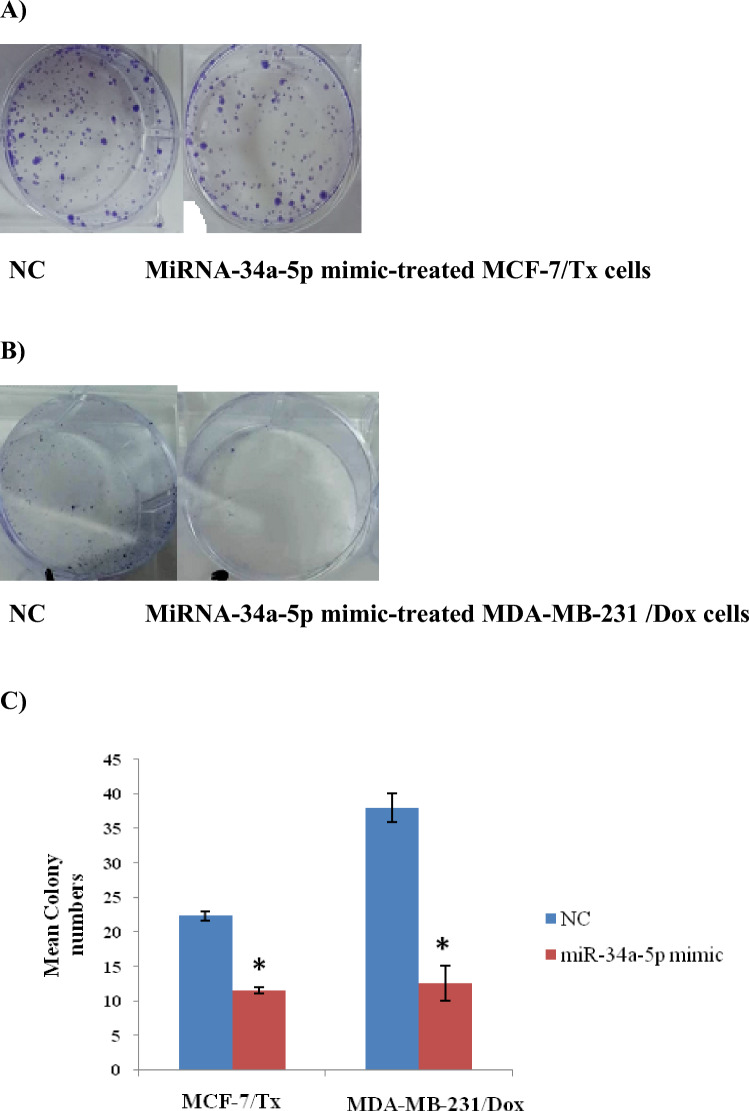
Colony formation in drug-resistant breast cancer cells treated with miRNA-34a-5p mimic/NC. **A** MCF-7/Tx cells**; B** MDA-MB-231/Dox cells**; C** A graphical chart of mean colony numbers for both cells as compared to NC, data were represented as mean±SEM, *P < 0.05

**Fig. 6:**
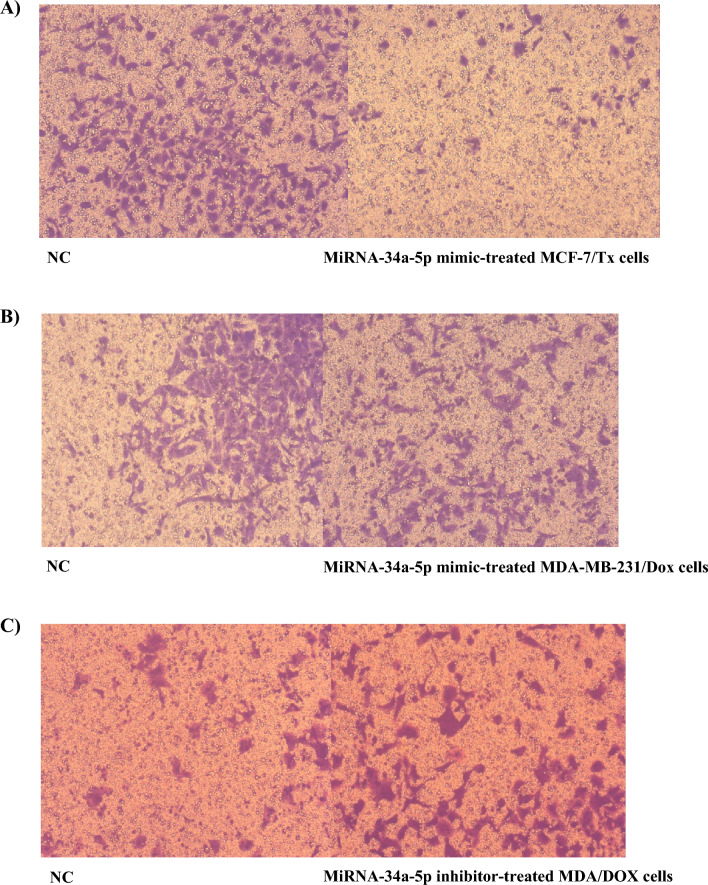
Migration inhibition of MCF-7/Tx and MDA-MB-231/Dox cells in response to exogenous manipulation of miRNA-34a-5p expression. **A**, and **B** Representative images of migrated cells after transfection of MCF-7/Tx, and MDA-MB-231/Dox cells with miR-34-a-5p mimic for 48 h using transwell migration assay, respectively, and **C** The effect of miRNA-34a-5p inhibitor treatment on migration in MDA-MB-231/Dox cells

**Fig. 7:**
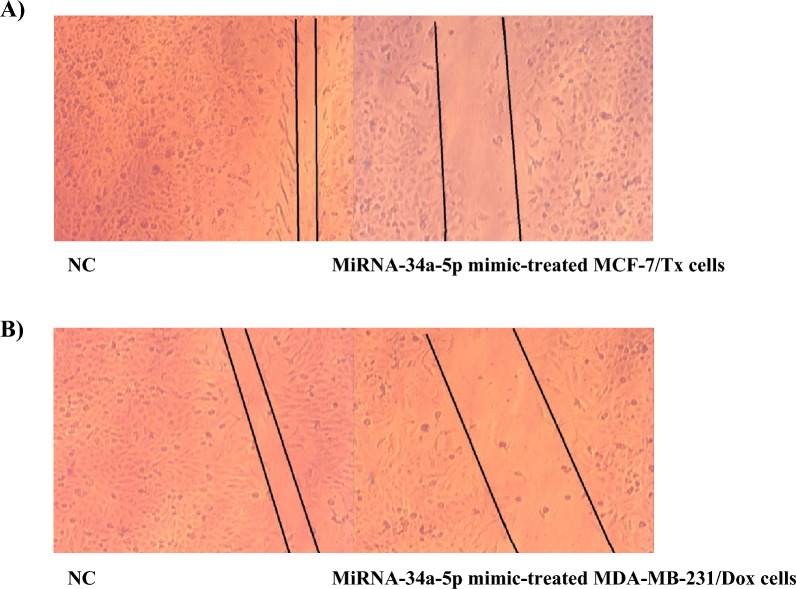
Wound healing assay. **A** MiRNA-34a-5p mimics transfected MCF-7/Tx cells, and **B** MDA-MB-231/Dox treated with miRNA-34a-5p mimic. Photos were captured 24 h after creating the scratch

To investigate the anti-proliferative therapeutic function of miRNA-34a-5p restoration in drug-resistant breast cancer cell lines, MCF-7/Tx and MDA-MB-231, colony formation, transwell migration, and wound healing assays were performed. Results of the colony formation (Fig. 5), the migration ability (Fig. 6 and Table 4), and wound healing (Fig. 7) showed a significant attenuation of metastatic potential and cellular growth suppression upon miRNA-34a-5p upregulation in both types of breast cancer-resistant cells.

**Table 4:** Relative migration percentage as compared to NC-treated cells

Treatment	Change in migration percentage relative to control cells
MCF-7/Tx	
NC	100%
MiRNA-34a-5p mimic	22%
MDA-MB-231/Dox	
NC	100%
MiRNA-34a-5p mimic	85%
MiRNA-34a-5p inhibitor	243%

### The apoptotic and necrotic activities of miRNA-34a-5p

**Fig. 8:**
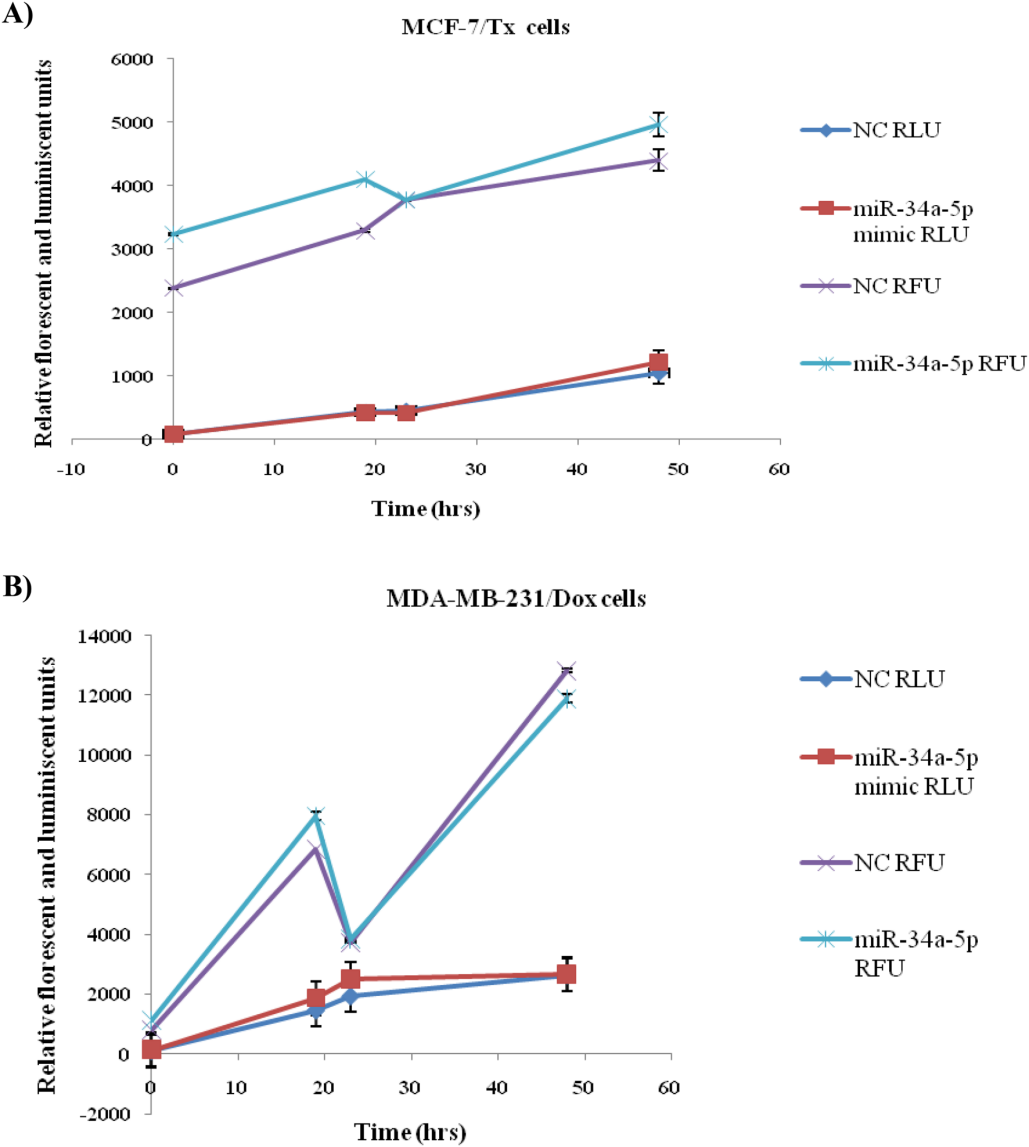
Annexin assay for determination of apoptosis and necrosis. **A** MCF-7/Tx cells transfected by miRNA-34a-5p mimic/NC, and **B** MDA-MB-231/ Dox cells treated with miscript miRNA-34a-5p negative control (NC), miRNA-34a-5p mimic. *RLU* Relative Light Unit, and *RFU* Relative Fluorescent Unit. Data were presented as mean ± SEM

**Fig. 9:**
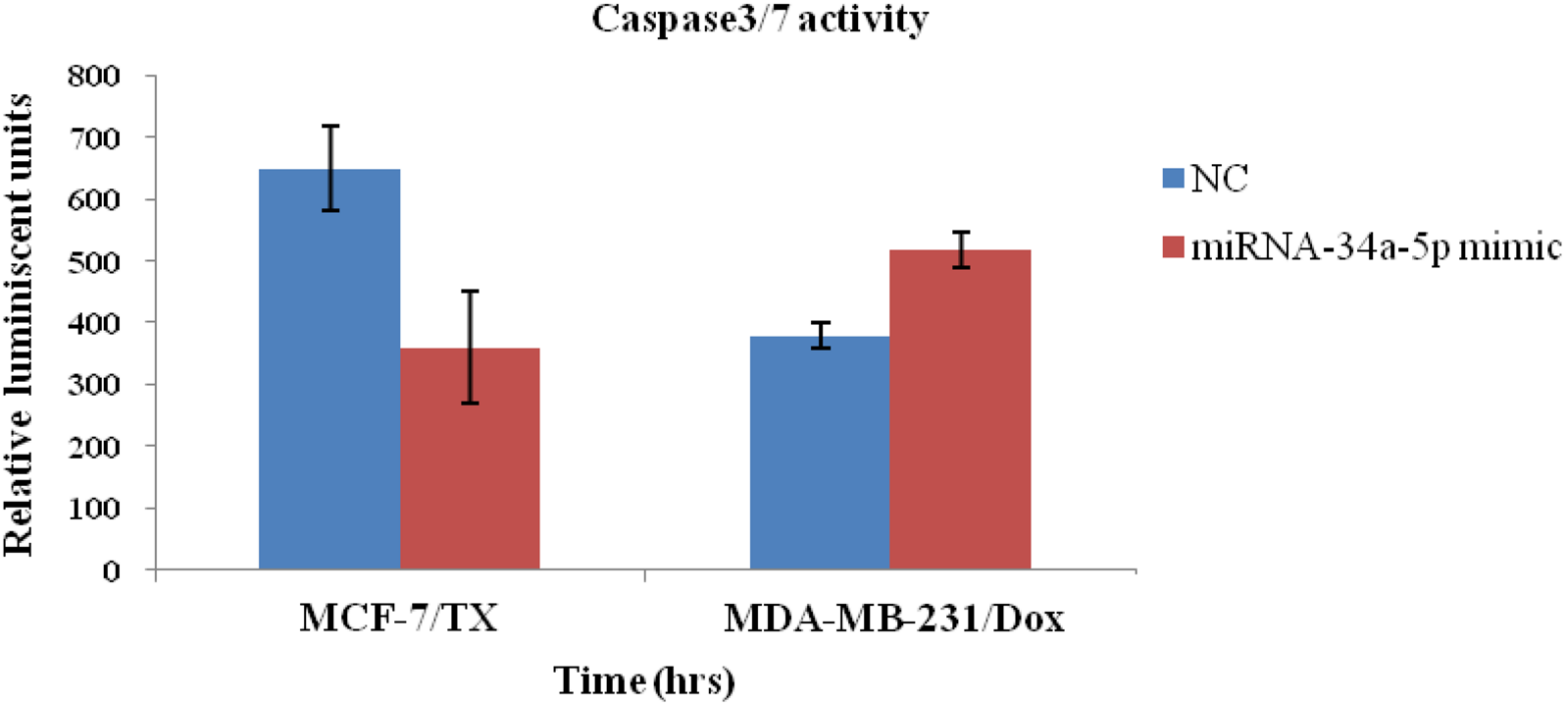
Caspase3/7 activity in MCF-7/Tx and MDA-MB-231/Dox cells treated with miRNA-34a-5p mimic. Data were presented as mean ± SEM)

In the current study, both annexin apoptosis assay and caspase-3/7 activity didn’t confirm a remarkable effect of miRNA-34a-5p restoration on apoptosis (Figs. 8 and 9). In annexin assay, miRNA-34a-5p mimic-treated cells (MCF-7/Tx and MDA-MB-231/Dox) produced time- and dose-dependent upregulation in luminescence which was concurrent with increased inflorescence which did augment neither apoptotic nor necrotic effects and suggested the involvement of alternative mechanisms of programmed cell death.

## Discussion

Breast cancer (BC) is the main cause of cancer mortality among women globally. According to statistics, BC accounts for around 23% of all cancer cases in women, and death due to breast carcinoma accounts for approximately 14% of all cancer-related mortalities in women, while the number of BC patients is increasing at a rate of more than 1.3 million per year. The poor response to chemotherapy due to the development of cellular drug resistance remains a key clinical obstacle to successful BC treatment [37, 38].

One of the most important mechanisms of drug resistance is drug elimination through the ATP-binding cassette (ABC) transporter family, which reduces intracellular drug accumulation by working as a pump rapidly extruding exogenous chemicals out of cells. The ABC family members including *ABCB1*, *ABCC1*, *ABCC2*, and *ABCG2* were also expressed to overlap in substrate recognition, making those transmembrane transporters one of the leading causes of medication failure [19].

MiRNAs play an important role in targeting specific genes related to chemotherapeutic resistance, and multiple of them target several genes contributed to different attributed biochemical pathways. Various studies have shown that rebalancing miRNA expression in resistant cells might re-sensitize cancer cells to anti-tumor therapies, bolstering the potential of miRNAs as co-adjuvants in anticancer treatments. MiRNA-34a, the master regulator of tumor suppression, was discovered to be downregulated in a variety of malignancies, including BC [6, 32]. Furthermore, miRNA-34a-5p, derived from miRNA-34a, has been perceived to inhibit cell migration, invasion, and tumor development [14] and suggested a correlated mechanism between miRNA-34a and anti-apoptotic proteins [39]. The strategy of miRNA-34a replacement has been explored in clinical trials as the first challenge of miRNA application in cancer treatment [40]. Therefore, the current research paper firstly underlined the potential of miRNA-34a-5p upregulation as a drug resistance modulator in established resistant BC cell lines and studied the anticancer therapeutic effects of miRNA-34a-5p restoration in MCF-7/ tamoxifen and MBA-MB-231/doxorubicin-resistant BC cells. Interestingly, our findings indicated that the overexpression of miRNA-34a-5p increased the sensitivity of resistant cells in a mechanism that is dependent on direct targeting the drug transporter *ABCC1* gene, as confirmed by dual-luciferase reporter assay, QRT-PCR, and influx assay. Moreover, the transfection of miRNA-34a mimic led to diminishing the metastatic and invasive properties of resistant BC cells, as observed by colony formation, transwell migration, and wound healing assays. However, our results suggested that there might be more active anti-proliferative mechanisms than apoptosis and cell cycle arrest.

An agreement with our data, He et al. [18] and Shao et al. [41] declared that miRNA-34a improves the sensitivity of cervical cancer cells toward oxaliplatin by targeting Murine Double Minute 4 (*MDM4)* expression and 5-fluorouracil by targeting lactate dehydrogenase A (*LDHA*), a glycolysis key enzyme, respectively. Moreover, overexpression of hsa-miRNA-34a-5p in gastric cancer SGC-7901/5-fluorouracil-resistant cells was found to decrease migration and invasiveness, as resulted by wound healing and transwell invasion assays, after chemotherapy. Likewise, hsa-miR-34a-5p enhanced the chemotherapy sensitivity of resistant gastric cancer cells by targeting *SIRT1*, *ABCB1*, and *ABCC1* [42]. However, the current results showed a non–significant reduction in luciferase activity in the case of *ABCB1* and *ABCC5* 3’UTR vector co-transfection with hsa-miR-34a-5p mimic. In addition, overexpression of hsa‑miRNA‑34a was detected to inhibit cell migration and invasion in bladder cancer cell lines 5637 and UMUC3 as detected by transwell assay, through matrix metalloproteinase-2 silencing [43]. Han et al. [33] confirmed that the treatment of T‑47D and MDA‑MB‑231 breast cancer cells with miRNA-34 mimic inhibited tumor cell proliferation, migration, and invasiveness as demonstrated by Colony formation, Wound healing, and transwell invasion assays. While upregulation of miRNA-34a in both these breast cancer cell lines increased the activity of caspase-3 via downregulation of stem cell-associated transcription factors, *E2F1* and *E2F3*. MiRNA-34a mimic was determined to inhibit the proliferation and colony formation of SKOV3 and OVCA433 ovarian cancer cells and enhanced cisplatin sensitivity of cisplatin-resistant SKOV3 ovarian cells via direct suppressing of *HDAC1* [44]. The ectopic expression of miRNA-34a in metastatic breast cancer cell-line BT-549 significantly inhibited cell migration and invasion by directly targeting epithelial to mesenchymal transition (EMT)-associated protein NOTCH1 [45].

More conformance oncology studies were reported in a research work conducted by Williams et al. [46] demonstrated that miRNA-34a mimic reversed cisplatin and vinorelbine resistance in malignant pleural mesothelioma cell lines and enhanced apoptosis. As well, upregulation of miRNA-34a resulted in attenuating drug resistance to 5-fluorouracil in colon cancer via downregulation of targets in the PI3K/AKT signaling pathway. Also, miRNA-34a restoration improved cisplatin sensitivity in bladder cancer cell lines via transcriptional suppression of *SIRT1* and *Cdk6*. Besides, increasing miRNA-34b expression enhanced susceptibility to gemcitabine, doxorubicin, and methotrexate, as well as triggered apoptosis. This was most likely accomplished by miR-34b modulation of *PAK1* and *ABCB1*, which are important regulators of the cell cycle, multidrug resistance (MDR), and apoptosis [46].

A study performed by Liu and co-workers [47] agreed with our observations. They detected that miRNA-34a mimic attenuated the paclitaxel-chemoresistance of PC-3PR prostate cancer cells through direct suppression of *JAG1* and *Notch1*expressions. Overexpression of miRNA-34a reduced the formation of prostate cancer cell clones as well. While high levels of miRNA-34a promoted cellular apoptosis and G0/G1 arrest of PC-3PR cells [47]. A direct interaction of miRNA-34a with the anti-apoptotic *BCL-2* gene was shown by luciferase assay in MCF-7, and MDA-MB-231 acquired docetaxel resistance [4]. Additionally, Liu and co-workers in 2012 suggested that the upregulation of miRNA-34a in prostate cancer cells was associated with the increased sensitivity of tumor cells to camptothecin and paclitaxel cytotoxic agents [38].

The mechanism by which miR-34a targets ATP-binding cassette (ABC) transporters could be explained by its direct targeting of the 3′UTR of the ornithine decarboxylase antizyme 2 (*OAZ2*), resulting in suppression of MDR-associated ABC transporters. This finding was reported earlier in colorectal adenocarcinoma where the ectopic expression of exogenous miRNA-34a re-sensitized MDR-human colorectal adenocarcinoma cell-line HCT-8/OR to oxaliplatin treatment through targeting *ABCB1*, *ABCC2*, *ABCG2* and antiapoptosis pathways [48].

More investigations conducted in doxorubicin-resistant MCF-7 breast cancer cells partially supported our conclusion. Ectopic overexpression of miRNA-34a in MCF-7/doxorubicin cells could sensitize the resistant cells to chemotherapy by directly targeting *Notch1*, resulting in prevention of cell invasion using transwell assay, and induced early and late apoptosis and G1 phase arrest, as compared to negative control [37, 38]. Also, miRNA-34a mimics were recorded to increase the sensitivity and death of MDR-MCF-7 cells by targeting *BCL-2*, *CCND1*, and *NOTCH1*. The flow cytometric analysis of cell cycle distribution estimated a higher percentage of cells stayed in the G2/M phase, and the proportion of cells in the G0/G1 phase was lower than that of negative control-treated cells [32].

Contrary to our conclusion, Pu et al. reported that miRNA-34a-5p mimic-restoration sustained the multi-chemoresistance of MDR- SJSA-1 osteosarcoma cell lines via direct repression of angiotensin II type 1 receptor (*AGTR1*) [49], Delta-like ligand 1 (*DLL1*), the ligand of the Notch pathway, [50], and receptor tyrosine kinase *CD117* [51].

Our results also showed an insignificant decrease in the percentage of cell population arrested in the S-phase (14.16 ± 0.23%) of the cell cycle as compared to negative control-treated MDA-MB-231/Dox cells (16.27 ± 0.96%). This observation was also confirmed by a study carried out by Niu et al. [52] using a model of human hepatocellular carcinoma SMMC7721 and MHCC97H cells.

Collectively, restoration of miRNA-34a-5p increases the sensitivity of the drug-resistant breast cancer cells via direct inhibition of the *ABCC1* gene which is confirmed to has a role not only in mediating chemotherapeutic drugs exportation, but also in transporting a variety of substrates involved in inflammation and cellular signaling and proved in several different types of cancer to have a role in proliferation, clonogenic capacity, cell migration, and invasion, such as bioactive lipids sphingosine-1-phosphate, and lysophosphatidyl inositol [53]. Additionally, hence doxorubicin mediates its cell death action through a number of proposed models, including topoisomerase II poisoning, DNA adduct formation, oxidative stress, and ceramide overproduction, which are dependent on the corresponding doxorubicin dose [54], the maintained doxorubicin concentration as detected by influx assay will intercede the comparable cell death action.

## Conclusion

In summary, the miRNA-34a-5p/ABCC1 axis has positive feedback effects on the sensitivity of MCF-7 and MDA-MB-231 cells to tamoxifen and doxorubicin, respectively, which is expected to be an important target for these chemotherapies in resistant breast cancer progression future studies. Totally, the current investigation introduced miRNA-34a-5p as positive regulators for ATP-binding cassette (ABC) transporter genes and oncogenic properties of drug-resistant breast cancer cells. MiRNA-34a could be used as a therapeutic molecule for reducing the developed drug resistance in breast cancer and the associated tumorigenic characteristics.

## Data Availability

All are available in the current research paper. For more required details, you could contact the corresponding author.

## Abbreviations

3′UTR: 3′- Untranslated regions
ABC: ATP-binding cassette
AGTR1: Angiotensin II type 1 receptor
ATCC: American Type Culture Collection
BC: Breast cancer
BCRP: Breast cancer resistance protein
DLL1: Delta-like ligand 1
DMEM: Dulbecco’s Modified Eagle Medium
DPBS: Dulbecco’s phosphate-buffered saline
EMT: Epithelial to mesenchymal transition
FBS: Fetal Bovine Serum
FLU: Florescent and luminescent unit
LDHA: Lactate dehydrogenase A
MCF-7/Tx: MCF-7/ tamoxifen
MDA-MB-231/Dox: MDA-MB-231/Doxorubicin
MDM4: Murine Double Minute 4
MDR: Multidrug resistance
MiRNA: MicroRNA
NC: Negative control
OAZ2: Ornithine decarboxylase antizyme 2
PBS: Phosphate-buffered saline
P-gp: P-glycoprotein
PI: Propidium iodide
RFU: Relative Fluorescent Unit
RLU: Relative Light Unit
SEM: Standard error of mean
